# Circulating tumor cells: potential markers of minimal residual disease in ovarian cancer? a study of the OVCAD consortium

**DOI:** 10.18632/oncotarget.22468

**Published:** 2017-11-16

**Authors:** Eva Obermayr, Natalia Bednarz-Knoll, Beatrice Orsetti, Heinz-Ulrich Weier, Sandrina Lambrechts, Dan Cacsire Castillo-Tong, Alexander Reinthaller, Elena Ioana Braicu, Sven Mahner, Jalid Sehouli, Ignace Vergote, Charles Theillet, Robert Zeillinger, Burkhard Brandt

**Affiliations:** ^1^ Department of Obstetrics and Gynecology, Medical University of Vienna, Vienna, Austria; ^2^ Institute of Tumor Biology, University Medical Center Eppendorf, Hamburg, Germany; ^3^ INSERM U1194, IRCM, Université de Montpellier, Montpellier, France; ^4^ Institut du Cancer de Montpellier, Montpellier, France; ^5^ Department of Cancer and DNA Damage Responses, Life Sciences Division, University of California, Lawrence Berkeley National Laboratory, Berkeley, CA, USA; ^6^ Division of Gynecological Oncology, Department of Obstetrics and Gynecology, Leuven Cancer Institute, University Hospitals Leuven, Katholieke Universiteit Leuven, Leuven, Belgium; ^7^ Department of Gynecology, European Competence Center for Ovarian Cancer, Campus Virchow Klinikum, Charité- Universitätsmedizin Berlin, Berlin, Germany; ^8^ Department of Gynecology and Gynecologic Oncology, University Medical Center Hamburg-Eppendorf, Hamburg, Germany; ^9^ Institute of Clinical Chemistry, University Medical Center Schleswig-Holstein, Campus Kiel, Kiel, Germany; ^10^ Department of Gynecology and Obstetrics, University of Munich, Munich, Germany

**Keywords:** circulating tumour cells, minimal residual disease, ovarian cancer, multi-marker analysis, FISH on CTCs

## Abstract

**Purpose:**

In 75% of ovarian cancer patients the tumor mass is completely eradicated by established surgical and cytotoxic treatment; however, the majority of the tumors recur within 24 months. Here we investigated the role of circulating tumor cells (CTCs) indicating occult tumor load, which remains inaccessible by established diagnostics.

**Experimental design:**

Blood was taken at diagnosis (baseline samples, *n* = 102) and six months after completion of adjuvant first-line chemotherapy (follow-up samples; *n* = 78). CTCs were enriched by density gradient centrifugation. A multi-marker immunostaining was established and further complemented by FISH on CTCs and tumor/metastasis tissues using probes for stem-cell like fusion genes MECOM and HHLA1.

**Results:**

CTCs were observed in 26.5% baseline and 7.7% follow-up blood samples at a mean number of 12.4 and 2.8 CTCs per ml blood, respectively. Baseline CTCs indicated a higher risk of death in R0 patients with complete gross resection (univariate: HR 2.158, 95% CI 1.111–4.191, *p* = 0.023; multivariate: HR 2.720, 95% CI 1.340–5.522, *p* = 0.006). At follow-up, the presence of CTCs was associated with response to primary treatment as assessed using RECIST criteria. Chromosomal gains at MECOM and HHLA1 loci suggest that the observed cells were cancer cells and reflect pathophysiological decisive chromosomal aberrations of the primary and metastatic tumors.

**Conclusions:**

Our data suggest that CTCs detected by the multi-marker protein panel and/or MECOM/HHLA1 FISH represent minimal residual disease in optimally debulked ovarian cancer patients. The role of CTCs cells especially for clinical therapy stratification of the patients has to be validated in consecutive larger studies applying standardized treatment schemes.

## INTRODUCTION

Ovarian cancer has the highest mortality rate of all gynecological cancers [1]. The standard of care for patients with advanced stage ovarian cancer consists of maximal cytoreductive or debulking surgery, and followed by a platinum-based adjuvant chemotherapy in combination with paclitaxel [2]. Residual disease after surgery is one of the most relevant prognostic factors for the patients [3]. However, even in optimally debulked patients without evidence of macroscopic residual tumor mass and/or with complete clinical response to first-line chemotherapy (according to the GCIG criteria [4]), the disease will recur in about 80% of these patients within 24 months, with fatal outcome.

Currently, there is a major effort to identify biological markers of minimal residual disease (MRD), which remains inaccessible by clinically established staging procedures and measurement of the serum tumor marker CA-125. In this regard, circulating tumor cells (CTCs) have been actively investigated in a number of solid tumor types, predominantly using the FDA approved CellSearch assay (Janssen Diagnostic, USA) [5]. In ovarian cancer, there has been growing evidence that CTCs are of prognostic relevance as well (reviewed by Romero-Laorden *et al.* [6]). Hematogenous spread has not been deemed a major issue for this type of cancer, until Pradeep and colleagues showed in a parabiosis mouse model that ovarian cancer disseminates to the omentum and subsequently to the peritoneum not only due to intraperitoneal “seeding”, but also via the hematogenous route [7]. Thus we hypothesized that CTCs may serve as indicators for tumor load at any time and for treatment response in ovarian cancer as well, and that CTCs could be used as surrogate markers of the risk of recurrence and metastasis.

The aim of the present study was to investigate the prognostic impact of CTCs on the outcome of ovarian cancer patients included into the OVCAD study cohort, and whether these CTCs could serve as surrogate markers for MRD beyond surgery. Initially, the blood samples were processed using a combined approach consisting of an immune-magnetic enrichment of CTCs expressing the epithelial cell surface marker epithelial cell adhesion molecule (EpCAM) and a subsequent immune-fluorescent staining of intracellular cytokeratins (protocol A). In the course of the study we developed a more comprehensive protocol for the immune-fluorescent multi-marker staining of additional targets, namely epithelial growth factor receptor (EGFR), human epidermal growth factor receptor 2 (HER2), mucin 1 (MUC1), as well as EpCAM and cytokeratins (protocol B). The blood samples were taken at two time-points: First, at primary diagnosis before any therapeutic intervention (baseline samples), and second, six months after completion of the adjuvant platinum-based chemotherapy (follow-up samples). In addition to immune-fluorescent staining of CTCs, we performed fluorescence *in situ* hybridization (FISH) in some selected cases to investigate the copy number of the stem-cell like fusion genes MECOM/HHLA1 and to pinpoint CTCs as cancer cells in case of doubt.

## RESULTS

### Patient characteristics

**Figure 1:**
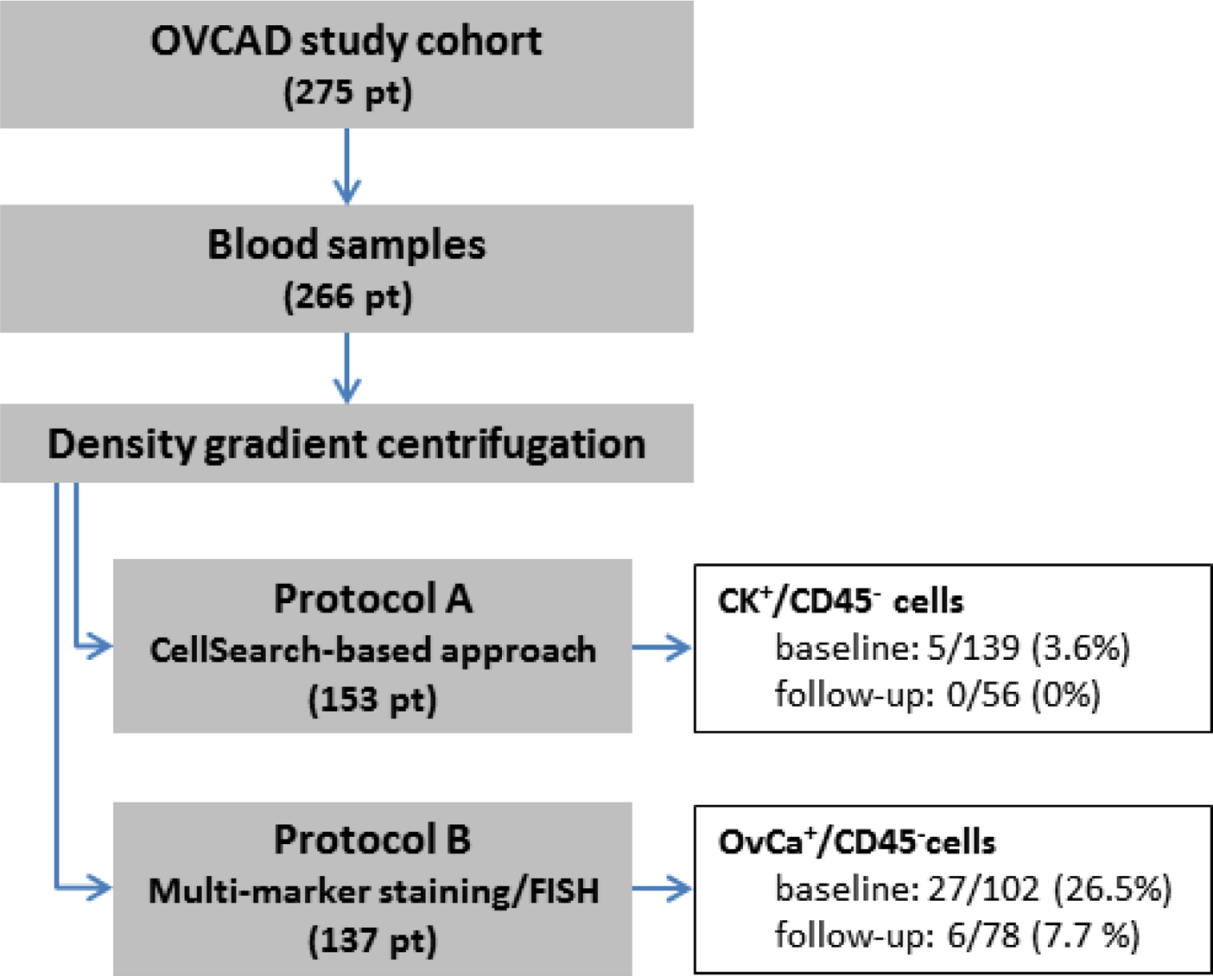
Flow diagram depicting the sample processing according to protocol A and B, including basic results

Blood samples were available from 266 of the 275 OVCAD study patients, comprising 241 samples taken at diagnosis, and 134 samples taken six months after completion of the primary treatment. The samples were processed according to protocol A in the first part of the study and to protocol B in the later course (see Figure 1). The patients were mainly diagnosed with FIGO stage III disease (77.1%), with high grade (72.2%), and serous (86.1%) histology. The majority of the patients (68.0%) was optimally debulked leaving no macroscopically visible tumor mass after surgery (R0); in 18.4% of the cases, neo-adjuvant chemotherapy was administered prior to surgery. Six months after completion of the adjuvant chemotherapy, 74.4% of the patients were classified as responders and 24.8% as non-responders according to the RECIST criteria. The proportion of FIGO stage IV patients was significantly higher in the protocol B than in the protocol A baseline samples (30.4% vs. 11.5%; *p* = 0.001), as well as the proportion of high grade tumors (79.4% vs. 64.7%; *p* = 0.022). Among the follow-up samples processed according to protocol A, we observed more R0 patients (82.1% vs 62.8%; *p* = 0.015) and patients having received a neo-adjuvant chemotherapy (26.8% vs. 12.8%; *p* = 0.046) than in protocol B follow-up samples. The median follow-up time of protocol B patients was 67 months (interquartile range, 61 to 73 months), with 108 recurrences and 83 cases of deaths.

### CK^+^/CD45^–^ CTCs in protocol A

In total, blood samples from 153 patients were processed according protocol A, which combined density gradient centrifugation, immune-magnetic isolation of EpCAM-positive cells and immune-fluorescent staining of cytokeratins. Blood samples taken at both time-points were available in 42 cases, whereas just one sample was available in (baseline) and in 14 (follow-up) cases, respectively. We observed CK^+^/CD45^–^ CTCs in 5/139 (3.6%) baseline samples and in none of the 56 follow-up blood samples [8] (see Figure 1). More than one CK^+^/CD45^–^ CTC was found in just a single baseline blood sample. The primary tumors of the five CTC-positive patients were classified as FIGO stage III (*n* = 4) and IV (*n* = 1). Due to the low number of CTC-positive samples, we did not perform any further statistical evaluation of the data.

### Additional CTC markers for protocol B

Based on an extensive literature research we selected EpCAM, EGFR, HER2, and MUC1 to complement the immune-fluorescent detection of CTCs based on cytokeratins alone in protocol A. To find out whether these markers were indeed appropriate to detect CTCs in our cohort, we evaluated the microarray gene expression data from the respective primary tumor tissues. These data, which were available from 93 of the 137 patients whose blood samples had not been processed according protocol A, confirmed the presence of the selected markers at the gene expression level in all tumor tissues samples; moreover, a complementary or additive expression was shown. The strongest positive correlations were observed between *EpCAM* and *KRT18*, between *KRT8* and *KRT18*, and between *KRT7* and *KRT8*. Furthermore, we observed a moderate correlation between *HER2* and *EpCAM*, and *KRT18*, respectively. Moderate to weak negative correlations were found between *MUC1* and *EGFR* and between *KRT7* and *KRT8* (see Table 1).

**Table 1:** Correlation between lg2-transformed and normalized gene expression levels of *EPCAM, KRT8, KRT18, KRT5, KRT7, HER2, MUC1,* and *EGFR,* resulting from microarray analysis of primary tumor tissue samples

	*EPCAM*	*KRT8*	*KRT18*	*KRT5*	*KRT7*	*HER2*	*MUC1*	*EGFR*
*EpCAM*	1							
*KRT8*	0.370**							
*KRT18*	0.654**	0.760**						
*KRT5*	-	0.335**	0.204*					
*KRT7*	-	0.635**	0.279**	0.353**				
*HER2*	0.441**	-	0.351**	-	-			
*MUC1*	0.372**	–0.240*	-	-	–0.510**	0.468**		
*EGFR*	-	–0.284**	-	-	–0.286**	0.314**	0.355**	1

Pearson’ correlation coefficients are given, *correlation significant at the 0.05 level, **correlation significant at the 0.01 level, - correlation not significant.

### OvCa^+^/CD45^–^ CTCs in protocol B and patient/tumor characteristics

Finally, in the second part of the study, we processed blood samples from 137 patients according to protocol B, which combined density gradient centrifugation and multi-marker immune-fluorescent staining targeting EpCAM, EGFR, HER2, MUC1, and cytokeratins. Blood samples from both time-points were available from 43 patients, whereas just one sample was available in 59 (baseline) and in 35 (follow-up) cases, respectively (see Figure 1). OvCa^+^/CD45^–^ CTCs were identified in 27/102 (26.5%) baseline blood samples and in 6/78 (7.7%) follow-up samples, at a mean count of 12.4 CTCs per ml blood at baseline (median 2.5, range < 1–187), and of 2.8 CTCs per ml blood in the follow-up samples (median 1, range < 1–10) [8]. A detailed description of the positive cases including disease stage and CTC counts is given in Table 3. In none of the ten healthy donor samples OvCa^+^/CD45^–^ cells were observed [Brandt B, Alpers I., EUTROC European Scientific Meeting 2011, Valencia, Spain].

At baseline, OvCa^+^/CD45^–^ CTCs were detected more frequently at a higher disease stage (FIGO II/III/IV *p* = 0.005), whereas at follow-up these CTCs were more likely in patients who were classified as non-responders to chemotherapy (*p* = 0.015; see Table 2).

**Table 2:** The presence of CTCs and their association to clinico-pathologic characteristics of the patients

	Baseline blood samples				Follow-up blood samples			
	*N*	CTC+ (%)	CTC– (%)	*p*	*N*	CTC+ (%)	CTC– (%)	*p*
**Total cases**	102	27 (26.5)	75 (73.5)		78	6 (7.7)	72(92.3)	
**Median age, yrs (SD)**		56. 0 (±10.4)	60.0 (±11.7)	0.268		62.0 (±7.7)	57.0 (±11.8)	0.279
**FIGO stage**				0.005				0.408
II	4	0 (0.0)	4 (100.0)		2	0 (0.0)	2 (100.0)	
III	67	12 (17.9)	55 (82.1)		62	4 (6.5)	58 (93.5)	
IV	31	15 (48.4)	16 (51.6)		14	2 (14.3)	12 (85.7)	
**Histology**				0.758				0.442
Serous	86	22 (25.6)	64 (74.4)		71	5 (7.1)	66 (92.9)	
Non-serous	16	5 (31.3)	11 (68.7)		7	1 (14.3)	6 (85.7)	
**Grade**				0.807				0.606
G1 or G2	21	6 (28.6)	15 (71.4)		17	2 (11.8)	15 (88.2)	
G3	81	21 (25.9)	60 (74.1)		61	4 (6.6)	57 (93.4)	
**Response to therapy**				0.708				0.015
yes	72	20 (27.8)	52 (72.2)		61	2 (3.3)	59 (96.7)	
no	29	7 (24.1)	22 (75.9)		16	4 (25.0)	12 (75.0)	
**Neo-adjuvant CT**				0.116				0.168
yes	17	7 (41.2)	10 (58.8)		10	2 (20.0)	8 (80.0)	
no	85	20 (23.5)	65 (76.5)		68	4 (5.9)	64 (94.1)	
**Residual disease**				0.724				0.188
yes	33	8 (24.2)	25 (75.8)		29	4 (13.8)	25 (86.2)	
no	69	19 (27.5)	50 (72.5)		49	2 (4.1)	47 (95.4)	
**Peritoneal carcinosis**				0.152				0.313
yes	67	20 (29.9)	47 (70.1)		49	6 (12.2)	43 (87.8)	
no	26	4 (15.4)	22 (84.6)		21	0 (0.0)	21 (100.0)	
**Ascites**				0.152				0.582
yes	67	20 (29.9)	47 (70.1)		54	5 (9.3)	49 (90.7)	
no	26	4 (15.4)	22 (84.6)		16	0 (0.0)	16 (100.0)	
**CA-125**				0.369				0.148
<35 U/ml	7	3 (42.9)	4 (57.1)		26	4 (15.4)	22 (84.6)	
≥35 U/ml	82	20 (24.4)	62 (75.6)		39	1 (2.6)	38 (97.4)	
**HE-4**				0.580				
<290 pM	42	12 (28.6)	30 (71.4)		n.a.			
≥291 pM	51	12 (23.5)	39 (76.5)				

Cytospin samples from density gradient enriched blood samples taken at diagnosis and six months after completion of first-line chemotherapy were evaluated for the presence of CTCs (OvCa^+^/CD45^–^ cells) using protocol B. Samples with “ambiguous” staining results were considered to be CTC-negative, unless chromosomal gain was detected by FISH analysis. n.a.: not assessed.

**Table 3:** Characteristics of CTC-positive patients

Patient ID	Disease stage	PFS (months)	OS (months)	OvCa^+^/CD45^–^ cells per ml blood	
				baseline	follow-up
H071	IV (liver, spleen)	16	58	187	n.a.
B151	IV (lung)	65	65	20	n.a.
B132	IV (LN)	14	45	11	n.a.
B159	IV (liver)	12	27	9	10
H076	IV (inguinal LN)	cPD	9	5	n.a.
B117	IV (inguinal LN)	13	37	4	n.a.
H079	IV (liver, LN)	§	14	4	n.a.
V046	IV (spleen)	12	44	3	n.a.
B131	IV (liver)	25	48	2	n.a.
B137	IV (inguinal LN)	70	70	2	0
B175	IV (inguinal LN)	8	14	2	n.a.
L177	IV (spleen)	11	35	2	0
B126	IV (PE)	15	28	1	0
H083	IV (spleen)	§	10	1	0
B171	IV (inguinal LN)	12	34	0	3
B111*	IV (PE)	22	44	0	n.a.
B120	IIIC	9	21	28	n.a.
V047	IIIC	10	36	14	n.a.
B135	IIIC	cPD	4	12	n.a.
B157	IIIC	44	44	4	n.a.
B146	IIIC	31	34	3	n.a.
B144	IIIC	21	37	1	n.a.
H073	IIIC	36	53	1	n.a.
H082	IIIC	32	53	< 1	n.a.
I020	IIIC	12	18	< 1	n.a.
L220	IIIC	15	56	1	0
V035	IIIC	13	69	0	1
L083	IIIC	11	25	n.a.	1
L141	IIIC	8	9	0	< 1
L227	IIIC	11	20	n.a.	< 1
B129	IIIA	21	39	2	n.a.
B168	IIIA	14	67	11	n.a.

31 patients were scored as CTC-positive due to the presence of OvCa^+^/CD45^–^ cells in their baseline and/or follow-up blood sample (protocol B). The stage of the disease at diagnosis according to the FIGO classification is given (site of distant metastases in brackets).

*One further patient (B111) was scored as CTC-positive due to chromosomal gain in 8q24.

n.a.: blood sample not available; cPD: clinical progressive disease at completion of adjuvant treatment; §: date of recurrence not assessed; LN lymph node; PE malignant pleural effusion.

The presence of OvCa^+^/CD45^-^ CTCs at baseline was associated with significantly lower levels of *KRT5* (mean lg2 transformed gene expression 1.12 vs. 0.10, *p* = 0.040) and *KRT7* (mean lg2 transformed gene expression 1.34 vs. 0.36, *p* = 0.034) gene expression in the corresponding tumor tissue samples. To assess whether the gene expression in the primary tumor tissue would predict the presence of CTCs, we performed a binary logistic regression analysis, including disease stage and the respective gene expression levels as continuous variables. The results indicated that advanced disease and low *KRT5* gene expression were independent predictors of baseline CTCs (FIGO: HR 4.410 95% CI 2.926–5.894, *p* = 0.009; 87 *KRT5*: HR 0.697 95% CI 0.336–1.058, *p* = 0.020).

Due to our tight criteria for CTC positivity, we additionally observed “ambiguous” cells, which were predominantly double-positive (OvCa^+^/CD45^+^), weakly stained (OvCa^+/–^/CD45^–^), or double-negative (OvCa^–^/CD45) with a tumor-like morphology [8]. These “ambiguous” cells were found in 16/102 (15.7%) baseline and in 9/78 (11.5%) follow-up samples. They were more likely in FIGO III than in FIGO IV stage disease (*p* = 0.031), and were mainly observed in chemo-responders (8/9 cases) [Brandt B, Alpers I., OVCAD Meeting 2009, Leuven, Belgium].

### OvCa^+^/CD45^–^ CTCs and patient survival

Within the entire cohort of 102 patients with baseline blood samples available, the presence of OvCa^+^/CD45^–^ CTCs at diagnosis did not have a significant impact on survival (OS: HR 1.564, 95% CI 0.923–2.652, *p* = 0.096; PFS: HR 1.167, 95% CI 0.699–1.948, *p* = 0.555). In those 78 patients with a follow-up blood sample available, the presence of OvCa^+^/CD45^–^ CTCs at that time-point was associated with worse outcome (OS: HR 3.305, 95% CI 1.386–7.880, *p* = 0.007; PFS: HR 5.671, 95% CI 1.560–20.618, *p* = 0.008).

### OvCa^+^/CD45^–^ CTCs and minimal residual disease

**Figure 2:**
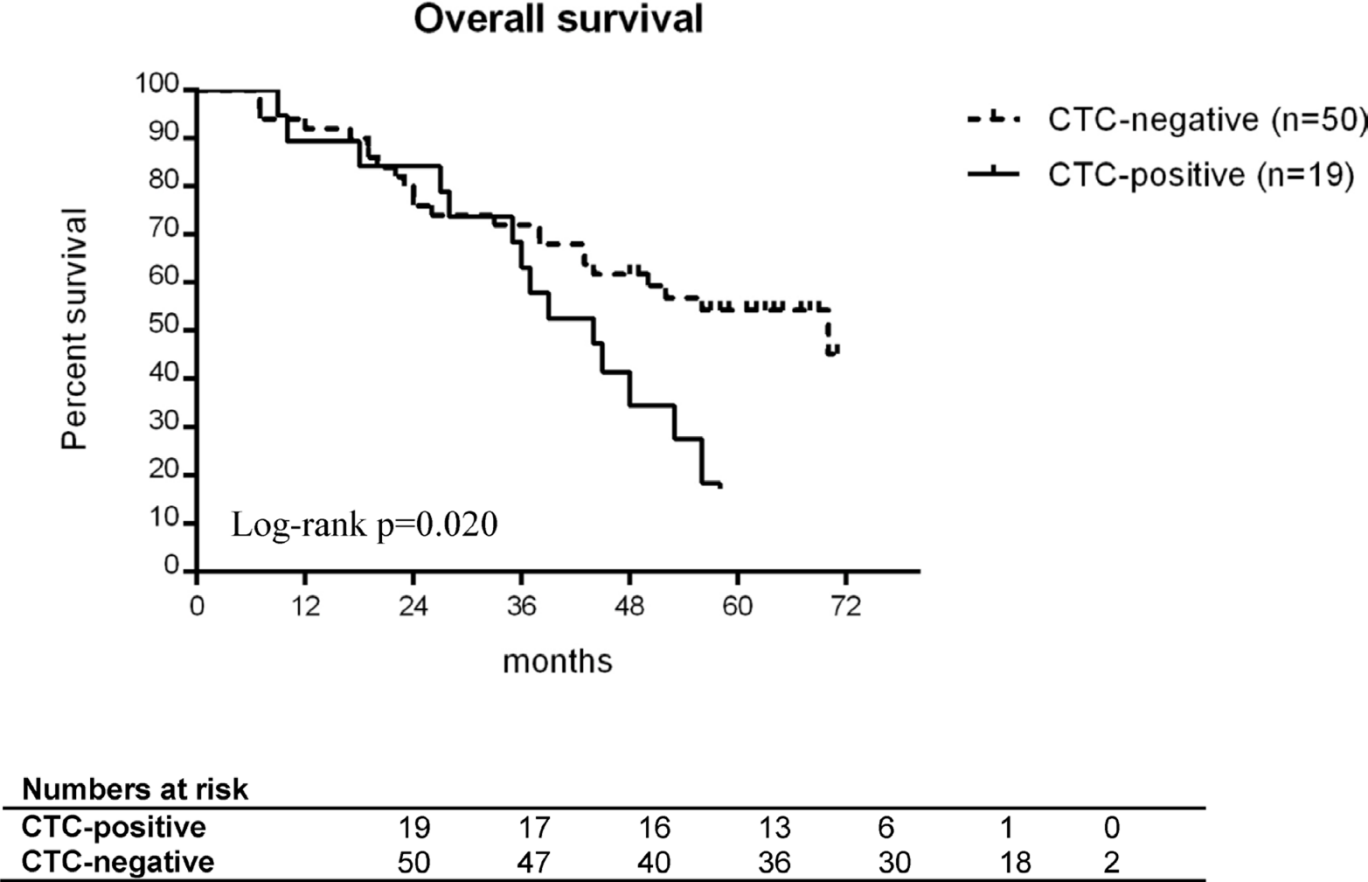
Overall survival of optimally debulked ovarian cancer patients The patients are stratified by presence (black line) or absence (grey line) of OvCa^+^/CD45^–^ CTCs at diagnosis. All patients (*n* = 69) received primary surgery without any macroscopically visible tumor residue left. Differences in survival were compared using the log-rank test.

To investigate the potential role of MRD after surgery, we stratified optimally debulked patients (R0) by the presence of OvCa^+^/CD45^–^ CTCs in their baseline blood samples. In these patients (*n* = 69), the presence of OvCa^+^/CD45^–^ CTCs at baseline was significantly associated with shorter OS (HR 2.158, 95% CI 1.111–4.191, *p* = 0.023) (see Figure 2), but not with shorter PFS. At the end of the total observation period, the majority of these CTC-positive patients (15/19) had already died, whereas about half of the CTC-negative patients (27/50) were still alive. According to multivariate analysis, OvCa^+^/CD45^–^ CTC-based outcome prediction was independent from patient age, FIGO stage, and the presence of peritoneal carcinomatosis (HR 2.720, 95% CI 1.340–5.522, *p* = 0.006).

### FISH analysis to confirm and complement CTC phenotyping

**Figure 3:**
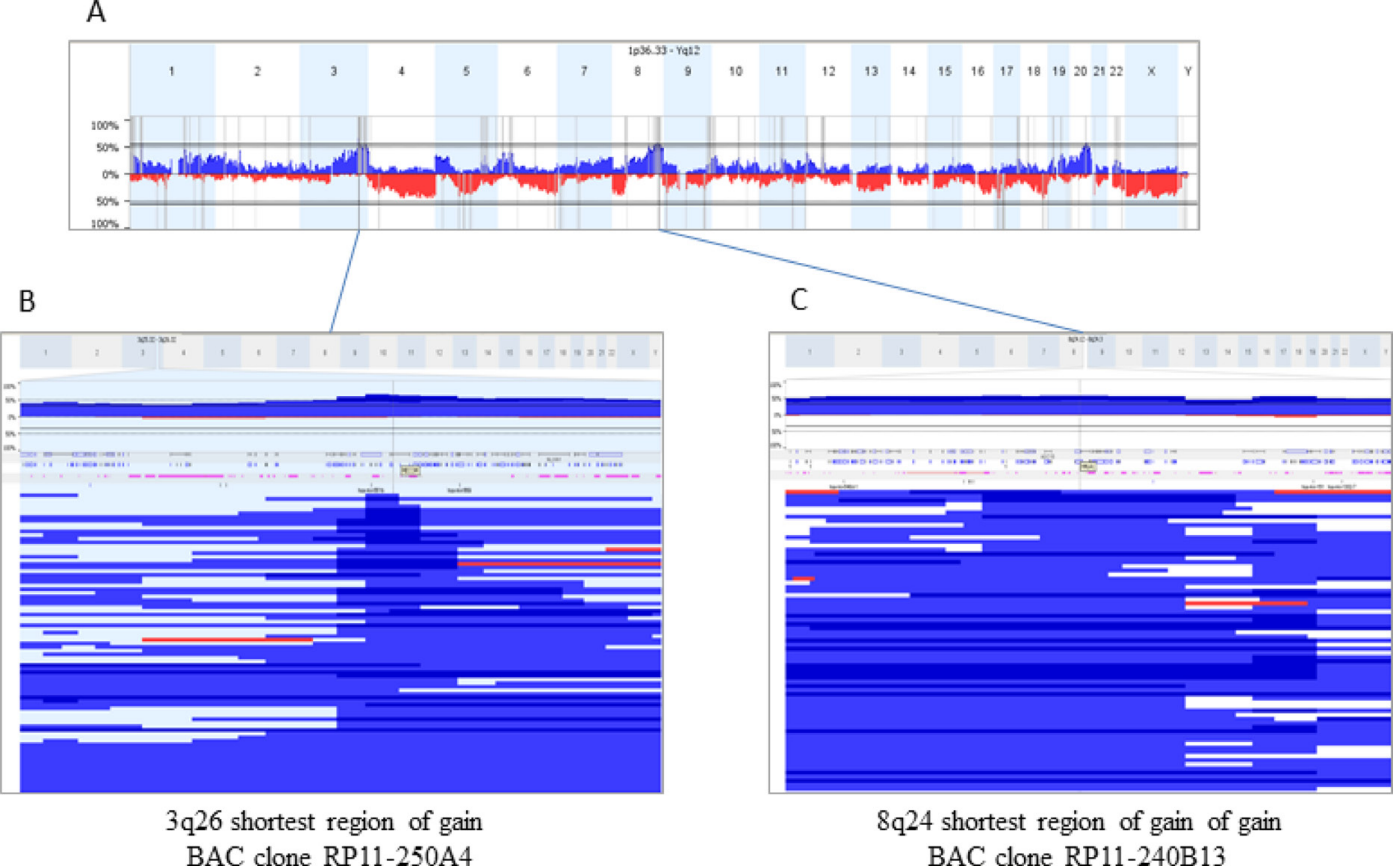
Copy Number Alterations (CNA) in 128 sporadic ovarian carcinomas and mapping of shortest regions of overlap (SRO) for gains at 3q26 and 8q24 (**A**) represents a whole genome CNA profile (gains blue, losses red). Regions at 3q26.2 and 8q24 were the most frequently gained (65.6 and 57% of the cases respectively). To select BAC best adapted BAC clones for FISH experiments, SROs were mapped (**B** and **C)** corresponding to blow up boxes showing profiles of all the tumors gained in the analyzed regions). BAC clones selected were RP11-250A4, at 3q26.2 covering the MECOM locus, and RP11-240B13, at 8q24 encompassing the HHLA1 gene.

CGH profiling had been performed as part of a preliminary study revealing gains at 3q26.2 and/or at 8q24 in 65.6% and 57% of the cases, respectively (Figure 3). The smallest region of overlap of gain at 3q26.2 corresponded to the MDS1/EVI1 (MECOM) locus, while that at 8q24 corresponded to the HHLA1 (POU5F1) locus (Figure 3). BAC clones RP11-250A4 and RP11-240B13 covering the MECOM and HHLA1 fusion loci were subsequently used as probes in FISH experiments in order to confirm the presence of cells related to progressive disease and possibly to metastasis.

A total of 1500 (RP11-240B13) and 3500 (RP11-250A4) leukocytes from three and seven healthy donors, respectively, were scored for gains of the FISH probes. The false-positive hybridization rate (three dots per cell) was 0.1% (RP11-250A4) and 0.5% (RP11-240B13), with more than 98% of the healthy donor leukocytes showing exactly two hybridization dots per cell [8]. In none of these leukocytes we detected more than three dots. Thus, we determined a cut-off value of at least three hybridization spots per cell to confirm that this cell was a tumor cell [8].

For FISH analysis of ovarian cancer CTCs, nine patients with more than two OvCa^+^/CD45^–^ CTCs per ml blood with three of them having “ambiguous” cells were selected. We found cells with MECOM and/or HHLA1 gains in all of the investigated blood samples (Table 4). Likewise, the detection of chromosomal gains complemented CTC phenotyping in those cases showing “ambiguous” double-negative cells (OvCa^–^/CD45^–^) with a tumor-like morphology and weakly stained cells (OvCa^+/–^/CD45^–^).

**Table 4:** Characteristics of FISH-positive patients

Patient	FIGO	OvCa^+^/CD45^–^ cells/ml blood	CNV (gains)	Time-point
H071	IV (liver, spleen)	187	MECOM/HHLA1	baseline
B120	IIIC	28	MECOM	baseline
B151	IV (lung)	20	MECOM/HHLA1	baseline
V047	IIIC	14	MECOM/HHLA1	baseline
B146	IIIC	3	MECOM	baseline
B175	IV (PE)	2	MECOM	baseline
L177	IV (spleen)	2	MECOM	baseline
B111*	IV (PE)	0	HHLA1	baseline
B159	IV (liver	10	MECOM	follow-up

Nine patients were selected for FISH analysis due to the presence of at least two OvCa^+^/CD45^–^ cells per ml blood.

*One further patient (B111) was scored as CTC-positive due to chromosomal gain in 8q24.

CNV copy number variation; PE pleural malignant effusion.

### MECOM and/or HHLA1 gains are associated with metastatic spread

In addition to the observation that all patients with primary metastasis (stage FIGO IV) presented with MECOM and/or HHLA1 positive CTCs, a phenotypical characterization of the OvCa^+^/CD45^–^ CTCs observed in a particular patient was performed. The baseline blood sample of that patient (H071) was the one with the highest number of CTCs (187 OvCa^+^/CD45^–^ cells per ml blood; see Table 3). These OvCa^+^/CD45^–^ cells were CK7/18-positive, with just 10% being EpCAM-positive, albeit at much lower staining intensity [8]. That difference of intensity was observed in metastases located in the spleen and liver as well, suggesting higher CK7/18 than EpCAM protein expression in these samples. In contrast, CKs and EpCAM were detected at similar levels in the primary tumor tissue. At the genome level, gains in the MECOM and HHLA1 loci were observed in the primary tumor and the metastases, as well as in just 25% and 50% of the CK7/18-positive CTCs, respectively. In addition, chromosomal gains were found in CK-negative cells which would not have been identified as tumor cells by immune-fluorescent staining alone.

## DISCUSSION

In the present study we developed a workflow for the detection and characterization of CTCs in ovarian cancer blood samples. The low number of CTC-positive samples observed in protocol A and in comparable studies employing epithelial markers alone [9, 10] led us to modify the protocol accordingly. Being aware of the heterogeneity of the CTC population, we assumed that a multi-marker approach might be more successful in ovarian cancer as well. We thereupon chose multiple targets, namely EpCAM, CKs, EGFR, HER2, and MUC1 for the immune-fluorescent detection of CTCs in the remaining blood samples (protocol B), and the analysis of chromosomal gains in MECOM/HHLA1 loci as a complementary approach. Although our study may have some limitations, our approach led us to the key observation that an unfavorable prognosis of optimally debulked patients was associated with the presence of CTCs before surgery. This finding indicates that CTCs may serve as biomarkers for MRD in ovarian cancer, and that they could therefore have the potential to become a decisive factor in the clinical management of ovarian cancer patients.

In view of the low number of CTC-positive samples in protocol A (just 5/139 baseline samples and none of the 56 follow-up samples contained CK^+^/CD45^–^ cells), we then used protocol B in the further course of the study, which comprised markers associated with epithelial characteristics (EpCAM, CK8/18) as well as with metastatic progression (MUC1, EGFR, CK5/7 and HER2 [11–13]) in order to catch various subsets of CTCs displaying different phenotypes. Furthermore, we combined antibodies from several sources to detect diverse epitope subsets [14]. In addition to the whole genome expression data from the OVCAD study cohort (data not shown), previously published studies employing the AdnaTest (Qiagen) indicated that MUC1, HER2, and EGFR could be suitable to complement epithelial markers. The AdnaTest is based on the immune-magnetic isolation of ovarian cancer CTCs using antibodies against MUC1 and EpCAM. On the molecular level, the majority of those CTCs were characterized by MUC1 and HER2 gene expression [15, 16].

In regard to the rareness and the heterogeneity of CTCs in ovarian cancer [17], we performed a density gradient centrifugation-based enrichment of up to 20 ml of blood as the very first step in both protocol A and B. Thereby we were able to analyze more than twice as much blood as other investigators using immune-magnetic bead-based techniques to enrich CTCs, such as the CellSearch assay (Janssen Diagnostic, USA) and the AdnaTest (Qiagen), which start from a 7.5 ml and 5.0 ml blood sample, respectively. The mean recovery rate of the above mentioned density gradient centrifugation was 76.6% for spiked-in ovarian cancer cells (data not shown), which is slightly lower than with prostate [18] or breast cancer cells [19]; nevertheless in our hands the same type of enrichment had already been proven to be effective for ovarian cancer and subsequent detection of CTC-related transcripts [20].

We observed noticeable more CTC-positive cases after switching from protocol A to B. By using multiple markers for the immune-fluorescent detection of CTCs, the numbers of positive cases increased from 5/139 (3.6%) to 27/102 (26.5%) at baseline, and from 0/56 (0%) to 6/78 (7.7%) at follow-up. In line with other studies, the presence of OvCa^+^/CD45^–^ CTCs in the protocol B samples significantly correlated with disease stage and therapy response [21–24].

However, the positivity rates obtained with protocol A and B may not be directly compared, because the respective blood samples differ in terms of patient characteristics, like disease stage and tumor grade. Thus the divergent results obtained with protocol A and B may only be partially attributed to the methodological differences of the respective protocols.

Interestingly, we observed that the presence of OvCa^+^/CD45^–^ CTCs detected in protocol B was associated with low *KRT5* and *KRT7* gene expression levels in the paired primary tumor tissue samples. Pan-cytokeratin antibodies are widely used for immune-histochemical staining of CK-positive tumor cells; however, there is sparse information available on cytokeratin phenotyping. Among the few studies investigating the role of CK5 protein in ovarian cancer, various rates of CK5-positive tumor tissue samples, ranging from 25% to 68%, are reported [25, 26]. Another study evaluating cytokeratins as biomarker to discriminate primary from metastatic adenocarcinoma found that CK7 protein expression is reduced in metastatic ovarian cancer [27]. Developing a gene expression profile that may predict the presence of CTCs–in line with Molloy’s study in breast cancer [28]–was out of the scope of the present study, but may eventually be subject of further investigations.

Studies investigating CTCs in ovarian cancer published to date vary markedly with regard to patient population, timing of blood draw, and length of follow-up. Furthermore, the methods for processing the blood samples and the decisive criteria for CTC-positivity vary greatly. For this reason any comparison of the results may only be carried out with caution. However, our results clearly support the notion of other authors that CTCs are present in the blood of ovarian cancer patients and that they may have clinical relevance (reviewed by [6]), and that in ovarian cancer a more comprehensive approach that does not rely on the detection of epithelial markers alone may be needed [20, 29–31].

The present results confirm the findings from our earlier study, which employed a combination of density gradient-based enrichment and detection of CTC–related gene markers using qPCR [20]. In both studies we demonstrated the prognostic impact of CTCs after chemotherapy; however, due to the limited sample number in the present study we were not able to assess whether that impact was independent from other known prognostic factors, and whether these CTCs may predict MRD in patients with clinical complete remission following treatment.

Our observation that the presence of baseline CTCs is associated with worse outcome in a sub-group of optimally debulked patients is in line with the findings of Pearl *et al.*, who used a cell adhesion matrix-based, functional cell enrichment platform to isolate invasive CTCs [24]. Like our approach to enrich CTCs by density gradient centrifugation, that platform may be regarded as being independent from epithelial markers; however the results obtained from the study mentioned above differ from those of our study in terms of median numbers of CTCs in ovarian cancer patients (i.e. 42 per ml vs. 2.8 per ml), and in terms of CTC presence in healthy and benign donors, which was observed at a rate of 5% in the Pearl study. These differences indicate that in our study some samples may be false negative. The presence of “ambiguous” double-negative cells further supports that assumption. We observed these “ambiguous” cells in 16% of the baseline and in 12% of the follow-up samples. In particular the OvCa^–^/CD45^–^ cells were discovered by chance, and it was out of the scope of our study to screen all samples for morphologically atypical and entirely unstained cells. A possible explanation for the decrease or even absence of a specific staining may be a reduced protein expression of the chosen markers or a proteolytic cleavage of their extracellular domains, both phenomena to occur during cancer progression [32–34]. Inconsistent results regarding CTC positivity rate and their prognostic relevance in earlier studies employing EpCAM-based systems may be at least partially due to the heterogeneity of the CTC population [35–37].

The presence of “ambiguous” cells demanded for an additional approach to ascertain whether these cells were cancer cells or not. Therefore, we analyzed the chromosomal aberrations in loci 3q26 and 8q24 using FISH probes for the survival and stem cell-like fusion genes MECOM and HHLA1. In concordance with earlier studies [38–40] we observed MECOM copy number gains in more than two thirds of the primary tumors of ovarian cancer patients. Likewise, it has been demonstrated that aberrations in the chromosomal region 8q24.21-8q24.22 comprising the HHLA1 locus occurred frequently in these cancers [41]. The presence of MECOM and/or HHLA1 gains in all CTC samples investigated confirmed the specificity of the multi-marker staining, and furthermore allowed us to ascertain that “ambiguous” cells corresponded to cancer cells, thus enabling us to extend the CTC count. In addition, we observed that even in patients with clearly assigned CTCs (OvCa^+^/CD45^–^ cells), a side population of unstained OvCa^–^/CD45^–^ cells harboring chromosomal gains was detected by FISH. Thus, in many ovarian cancer patients not only the actual number of CTCs but also their prevalence might be higher than assumed. The analysis of CTCs in patient H071 further revealed that these cells were heterogeneous not only regarding protein expression, but also at the genome level.

In conclusion, this study shows the prognostic impact of CTCs on the outcome of ovarian cancer patients included into the OVCAD study cohort. Our findings indicate that these CTCs could serve as surrogate markers for minimal residual disease beyond complete resection of the tumor. Future investigations using scanning microscopy or molecular probes may be useful in validating our preliminary findings in larger patient cohorts in order to strengthen the role of CTC diagnostics in the future management of ovarian cancer patients.

## MATERIALS AND METHODS

### Patients

The patients with histopathologically confirmed primary epithelial ovarian cancer were part of the prospective multi-center OVCAD study cohort. The overall goal of that study was to investigate new predictors for the early detection of MRD in epithelial ovarian cancer. Detailed inclusion and exclusion criteria, together with clinical data have already been presented elsewhere [42]. All patients received standard treatment consisting of debulking surgery and platinum-based adjuvant chemotherapy. Response to treatment was evaluated by experienced gynecological oncologists of the participating university centers according to the WHO criteria, i.e. by an increase in the nadir serum CA-125 level according to the GCIG criteria and by radiological (clinical) confirmation [43]. Patients were classified as non-responder if progression was diagnosed during treatment or recurrence occurred within six months after end of first-line chemotherapy. Written informed consent was obtained from each patient before study inclusion. The study protocol was approved by the local ethics committees of the participating OVCAD partners.

### Blood samples and CTC enrichment

Twenty ml of peripheral blood was taken by venipuncture at primary diagnosis (prior to treatment; i.e. baseline samples), and six months after completion of the adjuvant platinum-based chemotherapy (i.e. follow-up samples). The blood was collected into Vacuette EDTA tubes (Greiner Bio-One). A two-layer density gradient centrifugation to obtain a CTC enriched cell fraction and a plasma layer was performed as described previously [20]. Initially, the density gradient enriched cells were further processed employing protocol A, which finally was replaced by protocol B in the course of the study (see Figure 1). CA-125 and HE-4 levels were assessed in the top plasma layer obtained after density gradient centrifugation, using the Milliplex MAP Human Cancer Biomarker Panel Kit (Millipore) and HE-4 EIA assay (Fujirebio Diagnostics AB).

### CTC detection in protocol A

Following density gradient centrifugation as described above, the CTC containing cell fraction was further enriched for EpCAM-positive cells using the CellSearch Profile Kit as described by Alpers [7]. Then, each two cytospins were prepared (Hettich Rotofix 20 cytocentrifuge, 160x g, and 3 min), which were subsequently stained using fluorescently labelled antibodies to CK7/18 (clone A45-BB3, Micromet) and CK5/8 (clone C22, Progen). Leukocytes were counterstained using an Alexa Fluor 488 labelled antibody to hematopoetic lineage-specific CD45 (clone H130, Biolegend). Nuclei were visualized using DAPI (Carl Roth). CTCs were identified as CK^+^/CD45^–^ cells.

### Selection of CTC markers for protocol B

In light of the unexpected results obtained from samples which had been processed using protocol A in the initial phase of the study, we targeted EGFR, HER2, and MUC1 in addition to EpCAM and CK for the immune-fluorescent staining of CTCs in protocol B. These targets are known to be associated with cell differentiation, proliferation, tumor promotion and metastasis [11–13], and have already proven their suitability for CTC characterization [15, 16, 37]. Furthermore, microarray data from primary tumor tissue samples collected from the OVCAD cohort were available to verify the presence of these additional markers at the gene expression level [44].

### CTC detection in protocol B

After the enrichment by density gradient centrifugation, each three cytospins were prepared by applying a total number of 2 × 10^5^ enriched cells onto glass slides as described above. These samples were stained using a cocktail of primary murine antibodies to CK7/18 (clone A45-BB3, Micromet), CK5/8 (clone C22, Progen), EpCAM (clone Ber-EP4, Dako; clone VU-ID9, Novocastra), MUC1 (clone E29, Dako), EGFR (clone sc-120, Santa Cruz), and HER2 (clone NCL-CB11, Novocastra) as described by Alpers [7]. The appropriate dilution of each antibody to ensure high specificity was established using cell lines (MDA-MB-468, SK-OV3, MCF-7) and healthy donor leukocytes. Specific binding of the primaries was detected using a biotinylated secondary antibody (rabbit anti-mouse, Dako) and Alexa Fluor 594 labelled streptavidin (Invitrogen). Leukocytes and nuclei were visualized as described above. The specificity of multi-marker staining was assessed in the density gradient-enriched cell fraction of ten healthy donors. Unequivocal identification of CTCs (named further OvCa^+^/CD45^−^) in patients’ samples was allowed due to a strong Alexa Fluor 594 signal (OvCa^+^), the absence of leukocyte-specific staining (CD45^−^), and to the presence of an intact nucleus and cell morphology.

### BAC-based CGH analysis of tumor DNA

Comparative genomic hybridization (CGH) was performed on tumor DNA from 128 ovarian cancer patients, which was analyzed on two generations of BAC-arrays (Integragen) IgV6+ (5015 BACs), IgV7 (5878 BACs), with a median resolution of 0.6 Mb. BACs (bacterial artificial chromosomes) were spotted in quadruplicates. DNA labeling and hybridization were done as previously described [45] with slight modifications: 600 ng DNA were labeled with the BioPrime Total Genomic Labeling System (Invitrogen). Arrays were scanned using an Axon 4000B scanner (Molecular Devices), and images were analyzed using Genepix 6.0. Data were analyzed in a web-based platform for copy number array management and analysis (http://bioinfo-out.curie.fr/CAPweb/). Normalized and replicates-filtered data were exported as text file for further analyses. In order to analyze all the data from different Integrachip versions, we used the Nexus 6.0 Software (Biodiscovery). Analysis settings for data segmentation and calling were the following: significant threshold for Rank Segmentation algorithm: 0.005, Max Continuous Probe Spacing: 6000, Min number of probes per segment: 6, high level gain: 0.485, gain, 0.138, loss:−0.153, homozygous copy loss:−0.73. Nexus 6.0 Software was used to calculate frequency plots, factor enrichment (significantly over-represented factor values in a particular factor group identified using the two-tailed Fisher’s Exact test at a *p*-value of *p* < 0.05), significant chromosomal differences between two groups (two-tailed Fisher’s exact test with *p*-value < 0.005 and minimal frequency difference set at 10%) and Survival Predictive Power (log-rank test is used to identify genomic regions yielding a high degree of survival prediction; *p*-value was calculated by permuting the survival time for each sample and comparing the log-rank statistic for the permuted data to the original data; threshold used was *p*-value < 0.05).

### FISH on CTCs and primary tumor/metastasis tissues

The protocol was established as part of the doctoral thesis of Iris Alpers [8]. FISH probes were prepared from DNA isolated from BAC clones RP11-250A4 (3q26, MDS1 and EVI1 complex locus protein EVI1 (MECOM)) and RP11-240B13 (8q24, HERV-H LTR-associated 1 (HHLA1)) using the Large DNA Construct Isolation Kit (Qiagen) and the BioPrime Total Genomic Labeling System (Invitrogen) according to the manufacturers’ protocols. De-paraffinized FFPE sections and cytospin samples were processed as described previously [46] using 1 µl COT1 Human DNA (Roche), 1 μL of CEP7 Spectrum Aqua (Abbott Molecular) as a reference probe, and 2 μL of Spectrum Orange - labeled probe (Abbott Molecular) for chromosomal region 3q26 or 8q24 suspended in 6 μL of hybridization buffer. For each tissue specimen, target and reference probe signals were counted in 100 cells showing a minimum of two signals for the reference probe. DNA gain and loss were defined as the ratio of the number of target probe signal over reference probe signals being ≥1.5 or ≤0.75, respectively. For FISH on CTCs, the immune-staining protocol was slightly modified (streptavidin labelled with Alexa Fluor 488), and counterstaining of leukocytes was omitted. Leukocytes from healthy donors were scored for gains of the probes to obtain an estimate of the false-positive hybridization rate.

### Statistical analysis

The Pearson’s chi-square and Fisher’s exact test were used to assess the relationship between CTC presence and clinico-pathological characteristics of the patients. Clinical endpoints were calculated as follows: progression-free survival (PFS), between time of blood draw (baseline: prior to surgery, follow-up: six months after completion of adjuvant chemotherapy) and first recurrence; overall survival (OS), between time of blood draw (see above) and death due to any cause. Kaplan-Meier survival analyses and log-rank testing were used to compare survival outcomes [47]. Cox proportional hazards regression was used to determine univariate and multiple hazards ratios [48]. Covariates included were patient age as continuous variable, and FIGO stage (II/III vs. IV), residual tumor mass after surgery (R0 vs. R > 0), peritoneal carcinomatosis (absence vs. presence), and the CTC status (OvCa^+^/CD45^–^ absence vs. presence) as dichotomous variables. The model was built using a forward stepwise method by entering all variables at a *p* value of less than 0.05 and removing them at a *p* value of greater than 0.10. Statistical analysis was performed by SPSS version 19.0 (SPSS Inc., Chicago, IL). The level of significance was set at *p* < 0.05.

## Abbreviations

CTC: circulating tumor cell
GCIG: Gynecologic Cancer InterGroup
MRD: Minimal residual disease
CA-125: Cancer Antigen 125
HE-4: Human Epididymis Protein 4
FDA: US Food and Drug Administration
BAC: Bacterial artificial chromosome
FISH: Fluorescence *in situ* hybridization
EpCAM: Epithelial cell adhesion molecule
CK: Cytokeratin
EGFR: Epidermal Growth Factor Receptor
HER-2: Human epidermal growth factor receptor 2
CD45: cluster of differentiation 45
MUC1: Mucin 1
HHLA1: HERV-H LTR-Associating 1
MECOM: MDS1 And EVI1 Complex Locus
CNV: Copy number variation
HR: Hazard ratio
CI: Confidence interval
PFS: Progression free survival
OS: Overall survival
EIA: Enzyme-linked Immunosorbent Assay
FFPE: Formalin-Fixed, Paraffin-Embedded
